# Stability and Transformation of Metabolic Syndrome in Adolescents: A Prospective Assessment in Relation to the Change of Cardiometabolic Risk Factors

**DOI:** 10.3390/nu14040744

**Published:** 2022-02-10

**Authors:** Pei-Wen Wu, Yi-Wen Lai, Yu-Ting Chin, Sharon Tsai, Tun-Min Yang, Wei-Ting Lin, Chun-Ying Lee, Wei-Chung Tsai, Hsiao-Ling Huang, David W. Seal, Tsai-Hui Duh, Chien-Hung Lee

**Affiliations:** 1 Department of Public Health, College of Health Sciences, Kaohsiung Medical University, Kaohsiung 80708, Taiwan; catstar1211@gmail.com (P.-W.W.); rose8305072000@gmail.com (Y.-W.L.); kiki13336586@gmail.com (Y.-T.C.); fti32819@gmail.com (T.-M.Y.); 2 Department of Laboratory Medicine, Kaohsiung Municipal Hsiao-Kang Hospital, Kaohsiung 81267, Taiwan; 870718@kmuh.org.tw; 3 Department of Social, Behavioral, and Population Sciences, School of Public Health and Tropical Medicine, Tulane University, New Orleans, LA 70112, USA; wtlin0123@gmail.com (W.-T.L.); dseal@tulane.edu (D.W.S.); 4 Department of Family Medicine, Kaohsiung Medical University Hospital, Kaohsiung Medical University, Kaohsiung 80756, Taiwan; cying@ms19.hinet.net; 5 Division of Cardiology, Department of Internal Medicine, Kaohsiung Medical University Hospital, Kaohsiung Medical University, Kaohsiung 80708, Taiwan; azygo91@gmail.com; 6 Department of Internal Medicine, Faculty of Medicine, College of Medicine, Kaohsiung Medical University, Kaohsiung 80708, Taiwan; 7 Department of Oral Hygiene, College of Dental Medicine, Kaohsiung Medical University, Kaohsiung 80708, Taiwan; hhuang@kmu.edu.tw; 8 Department of Medicinal and Applied Chemistry, Kaohsiung Medical University, Kaohsiung 80708, Taiwan; 9 Research Center for Environmental Medicine, Kaohsiung Medical University, Kaohsiung 80708, Taiwan; 10 Department of Medical Research, Kaohsiung Medical University Hospital, Kaohsiung Medical University, Kaohsiung 80756, Taiwan; 11 Office of Institutional Research & Planning, Secretariat, Kaohsiung Medical University, Kaohsiung 80708, Taiwan

**Keywords:** metabolic syndrome, stability and transformation, cardiometabolic risk factor, anthropometric risk factors, latent clustering structure, cardiovascular risk, adolescent

## Abstract

Underlying pathophysiological mechanisms drive excessive clustering of cardiometabolic risk factors, causing metabolic syndrome (MetS). MetS status may transform as adolescents transition to young adulthood. This study investigated the latent clustering structure and its stability for MetS during adolescence, and assessed the anthropometric and clinical metabolic determinants for MetS transformation. A community-based representative adolescent cohort (*n* = 1516) was evaluated for MetS using four diagnostic criteria, and was followed for 2.2 years to identify new-onset MetS. The clustering structure underlying cardiometabolic parameters was stable across adolescence; both comprised a fat—blood pressure (BP)—glucose three-factor structure (total variance explained: 68.8% and 69.7% at baseline and follow-up, respectively). Among adolescents with MetS-negative at baseline, 3.2–4.4% had incident MetS after 2.2 years. Among adolescents with MetS-positive at baseline, 52.0–61.9% experienced MetS remission, and 38.1–48.0% experienced MetS persistence. Increased systolic BP (SBP) was associated with a high MetS incidence risk, while decreased levels of SBP and glucose were associated with MetS remission. Compared with adolescents with a normal metabolic status at baseline, those with an initial abdominal obesity and increased triglycerides level had a 15.0- and 5.7-fold greater risk for persistent abnormality, respectively. Abdominal obesity and low high-density lipoprotein cholesterol are two abnormal MetS components that highly persist during adolescence, and are the intervention targets for reducing the future risk of cardiometabolic disorders.

## 1. Introduction

The pathogenesis of cardiovascular disease starts in childhood, when cardiometabolic risk factors are first observed [1]. In adolescents, metabolic syndrome (MetS)—a syndrome involving the clustering of abdominal obesity, increased triglycerides (TG), high fasting plasma glucose (FPG), low high-density lipoprotein cholesterol (HDL-C), and elevated blood pressure (BP)—is a vital risk marker for future cardiometabolic disease [2]. Longitudinal studies have demonstrated that pediatric MetS is associated with a 2.0–2.9-fold risk for subclinical atherosclerosis, 2.3–11.5-fold risk for type 2 diabetes mellitus (T2DM), and 14.6-fold risk for cardiovascular disease after 14–30 years [3,4,5].

The clustering of cardiometabolic risk factors exceeds what should be observed for their accidental aggregation, indicating the presence of common underlying pathophysiological mechanisms [2,6,7]. Accordingly, the question of what cardiometabolic factor clustering structure reflects the latent pathogenic mechanisms and whether the clusters retain stable through growth stages in adolescents remains to be answered. An individual’s MetS status may change from adolescence to young adulthood [8,9,10]. One longitudinal study following adolescents up to adulthood revealed that 76.9% of the adolescents never had MetS, 16.4% had incident MetS, 5.7% experienced unstable/remitted MetS, and 1.1% had persistent MetS [9]. However, the change in MetS status and their stability during adolescence remains unclear. A comparison of MetS transformation using diverse MetS diagnostic criteria may help clarify this issue.

Prospective investigations have observed that 3.8–5.2% of MetS-negative adolescents (i.e., individuals aged 12–19 years) developed new MetS after 3 years. In contrast, 48.6–56.1% of MetS-positive adolescents achieved remission 3 years later, while MetS persisted in 43.9–51.4% of MetS-positive adolescents [8]. If the structure of cardiometabolic parameter clustering for MetS is stable over adolescent growth, an investigation into the effect of cardiometabolic risk determinants on the transformation of adolescent MetS is warranted. In cross-sectional studies involving adolescents, cardiometabolic risk factors have been observed to occur in childhood, but whether and to what extent they persist is unknown [11,12,13,14]. Insights into the persistence of cardiometabolic risk factors and how they contribute to the change of MetS can help create effective strategies for preventing MetS, increasing remission, and managing persistent MetS.

In a nationwide survey conducted in 2010–2011 in Taiwan, the prevalence of adolescent MetS, as defined by the Taiwan Pediatric Association (TPA) and International Diabetes Federation (IDF) diagnostic criteria, was 4.1% and 3.0%, respectively, with 22.1%, 12.3–19.3%, and 17.7–18.1% of adolescents having increased FPG, low HDL-C, and abdominal obesity, respectively [15]. Continuously monitoring and assessing the clustering of MetS risk factors and their impact on adolescent cardiometabolic health are warranted. This community-based longitudinal study investigated the latent clustering structure and its stability for MetS during adolescence, and evaluated the association of changes in anthropometric and clinical metabolic risk factors with the transformation of MetS over two years of follow-up.

## 2. Materials and Methods

### 2.1. Participants

The adiposity–cardiovascular disease axis (adi-Cars) investigation was a large representative cohort study conducted to investigate multilevel determinants and biomolecular risk profiles of cardiometabolic disease, prediabetes, and hyperuricemia among adolescents aged 12–14 years from southern Taiwan. The adolescents lived in three regions with varying levels of urbanization: Kaohsiung City, Pingtung County, and Taitung County. At the baseline survey, a three-stage procedure of random sampling of geographically stratified clusters was introduced to recruit the study participants. In stage one, Kaohsiung City, Pingtung County, and Taitung County were geographically stratified into nine, six, and four divisions, respectively. In stage two, all junior high schools within each division were compiled and listed, and 31 schools were randomly selected through computer-generated random numbers. In stage three, three classes (20–25 students per class) were randomly selected from each chosen school. One class was considered a cluster, and all students in the chosen classes were invited to participate in this study.

Baseline data were collected between September 2014 and June 2018, and follow-up data were measured between September 2017 and June 2021. A total of 2046 adolescents agreed to participate in the baseline anthropometric measurements and questionnaire surveys (response rate of 94.9%). Of these students, 1516 adolescents (74.1%) participated in the clinical biochemical examinations. In May 2021, a COVID-19 outbreak occurred in Taiwan and prevented follow-up by participants from three schools (these participants will be revisited after the outbreak). We excluded the students in these three schools in the data analyses. Eventually, 1246 adolescents from 28 schools with complete anthropometric and clinical blood data were followed. Of these, 1155 participated in the anthropometric and questionnaire survey at follow-up (retention rate: 92.7%; mean length of follow-up: 2.2 years), but only 896 participated in the clinical biochemical examinations. This study adhered to the principles expressed in the Declaration of Helsinki. The Institutional Review Board of Kaohsiung Medical University Hospital reviewed and approved this research project. Written assents from the adolescents and consents from their parents/guardians were collected for both the baseline and follow-up surveys.

### 2.2. Demographic and Cardiometabolic Factors

Multilevel-structured questionnaires were developed to obtain information on sociodemographic factors and lifestyle behaviors from adolescent participants and their parents. The urbanization of the township where each school is located was categorized into seven levels according to a socioeconomic cluster analysis of 359 Taiwan townships, with level 1 denoting the most urbanized [16]. Anthropometric parameters, including height, weight, hip circumference, waist circumference (WC), systolic BP (SBP), and diastolic BP (DBP), were measured at baseline and follow-up by a research team trained according to the World Health Organization guide for physical measurements [17]. Details of anthropometric measurements have been described previously [11,18,19,20,21]. Body mass index (BMI) was calculated as weight divided by height squared (kg/m^2^). Venous blood samples were obtained from participants in school health centers in the morning after a >10-h overnight fast. TG and HDL-C concentrations were enzymatically determined using a chemistry autoanalyzer and commercially available reagents, and the FPG levels were assessed using a glucose oxidase method (TBA-c16000, Toshiba, Tokyo, Japan) [22]. High-performance liquid chromatography (Bio-Rad Variant Turbo II HbA1c analyzer, Hercules, CA, USA) was used to measure the glycosylated hemoglobin (HbA1c) values.

### 2.3. MetS Diagnosis

MetS and MetS abnormal components were diagnosed using the IDF criteria for adolescents aged 10–18 years, TPA criteria for adolescents aged 8–18 years, and the Joint Interim Statement for adult MetS (JIS-Ad) [6,23,24]. Abdominal obesity was defined as WC ≥90th percentile (or adult cutoff if lower) by IDF, as BMI >95th percentile of age–sex-specific groups by TPA, and WC ≥90 cm in boys and WC ≥80 cm in girls by the JIS-Ad. Low HDL-C was defined as HDL-C <40 mg/dL for adolescents aged 10–15 years and HDL-C <40 mg/dL in adolescent boys and <50 mg/dL in adolescent girls aged 16–18 years by IDF. However, TPA and JIS-Ad define low HDL-C as HDL-C <40 mg/dL in boys and <50 mg/dL in girls. IDF, TPA, and JIS-Ad have the same criteria for increased TG (≥150 mg/dL), high FPG (≥100 mg/dL or previously diagnosed T2DM), and elevated BP (SBP ≥130 mmHg, DBP ≥85 mmHg, or antihypertensive drug treatment). Supplementary Table S1 presents the complete definitions for the five abnormal components given by IDF, TPA, and JIS-Ad. Because IDF and TPA have similar definitions for MetS abnormal components, we combined them as the IDF–TPA criteria to accommodate any outlier definitions of the IDF and TPA, and included all potential abnormal components. IDF-, TPA-, and IDF–TPA-defined MetS were the presence of abdominal obesity and any two other abnormal components. JIS-Ad-defined MetS was the presence of any three abnormal components.

### 2.4. Transformation of MetS Status

The IDF–TPA criteria for MetS and its abnormal components were used to investigate the transformation of MetS status over two years of follow-up in the adi-Cars cohort. Adolescents who were MetS-negative at both baseline and follow-up were defined as the never MetS group. Those who were MetS-negative at baseline but MetS-positive at follow-up were defined as the incident MetS group. Those who were MetS-positive at baseline and MetS-negative at follow-up were defined as the remitted MetS group. Those who were MetS-positive at both baseline and follow-up were defined as the persistent MetS group. The four groups were used as the main outcome of this investigation.

### 2.5. Statistical Analysis

We applied six statistical procedures for the data analysis. First, the demographic and cardiometabolic risk factors measured were analyzed as means ± standard deviation for the continuous variables and percentages for the categorical variables. Second, exploratory factor analysis (EFA) was employed to investigate the latent factor clustering structure across the cardiometabolic parameters for baseline and follow-up surveys, similar to a prior study [8]. Before performing EFA, all variables were assessed for Gaussian normality, and nonnormally distributed variables were converted using the logarithm function. Next, we performed Bartlett’s test of sphericity to examine whether cardiometabolic risk factors had a significant correlation structure. The Kaiser–Meyer–Olkin (KMO) measure of the sampling adequacy was used to evaluate the suitability of study data for structure detection. A KMO value of >0.50 was considered appropriate for the factor analysis. In EFA, we used principal component analysis as the factor extraction approach and applied the eigenvalue >1 rule, surpassing the break in scree plots to extract factors [25,26]. Varimax rotation was applied to obtain more interpretable factor loadings. The parameters with loadings >0.4 were used for interpreting the factors.

Third, Cohen’s Kappa coefficient (*k*) was calculated to evaluate the agreement of MetS (i.e., prevalence) defined by the four MetS criteria between baseline and follow-up surveys [27]. The *k* values of 0.21–0.40, 0.41–0.60, 0.61–0.80, and 0.81–1.00 were interpreted as fair, moderate, substantial, and almost perfect agreement, respectively [28]. Fourth, stratified by the group of MetS transformation, we applied a linear mixed model to assess within-person changes in the levels of cardiometabolic risk factors measured at baseline and follow-up [29]. Fifth, because quaternary outcomes (i.e., never, incident, remitted, and persistent MetS) were investigated, we used a multinomial logistic regression model to assess the association of changes in cardiometabolic risk factors with changes in MetS status over two years of follow-up. This modeling technique enables the simultaneous comparison of an outcome variable with >2 categories, and has been verified to have a higher precision and statistical power compared with simple binary outcome analysis [30,31]. An adjusted *p* value was calculated in order to correct for multiple testing using the false discovery rate controlling method [32]. Finally, incidence density was used to assess the status of new-onset MetS components in the follow-up survey. Multivariable Cox proportional hazards models and adjusted hazard ratios (aHRs) were applied to evaluate the association between initial abnormal status and subsequent abnormal occurrence for each MetS component. All of the multivariable models were adjusted for sex, age, urbanization level, and cardiometabolic risk factors, as appropriate. Statistical analyses were executed using statistical package Stata (StataCorp., College Station, TX, USA), version 17.

## 3. Results

Table 1 presents the distributions of demographic and cardiometabolic risk factors measured at baseline and follow-up for the adolescent cohort. The distributions of the sex and urbanization level for the participants were similar between the two time-points; age was obviously increased by two years. Compared with baseline, the adolescents had higher SBP, DBP, and weight-related variables and lower HDL-C and FPG at follow-up.

The study data on nine cardiometabolic risk factors collected at both baseline and follow-up had a significant correlation structure (Table 2; *p* for Bartlett’s test <0.001) and qualified for factor analysis (KMO; 0.782 and 0.794, both >0.5). EFA extracted three clustering factors from the study variables measured at both time-points (all factors, eigenvalues >1, and surpassing the break in scree plots; Supplementary Figure S1). The factor structures were similar at baseline and follow-up. Both comprised a fat factor (consisting of BMI, WC, hip circumference, HDL-C, and TG), a BP factor (consisting of SBP and DBP), and a glucose factor (consisting of FPG and HbA1c). The factor loadings for each factor and the proportion of variance explained by the three factors (68.8% and 69.7%, respectively) were very comparable between the data for the two time-points. All cardiometabolic risk factors used to interpret each factor structure had a factor loading of ≥0.508.

Table 3 displays the criterion-specific prevalence and transformations of adolescent MetS defined by four criteria over the two years of follow-up. The prevalence of MetS was 2.8–6.0% at baseline and 4.5–6.5% at follow-up, with a “fair level” of agreement (*k*, 0.313–0.367), indicating that MetS status changed with time (in this case, two years) in adolescents. Among the adolescents who were MetS-negative at baseline, 3.2–4.4% had incident MetS after two years. Among the adolescents who were MetS-positive at baseline, 52.0–61.9% experienced MetS remission and 38.1–48.0% experienced MetS persistence.

Table 4 presents within-person changes in cardiometabolic risk factors between baseline and follow-up, stratified by MetS typology per the IDF–TPA criteria. In the never group, BMI and WC at follow-up were higher and HDL-C and FPG were lower than the corresponding values at baseline. In the incident group, the within-person changes in BMI, WC, SBP, DBP, and HDL-C were significant and greater than those in the never group. In the remission group, the TG and FPG levels were noticeably reduced at follow-up. In the persistent group, the values of cardiometabolic risk factors were relatively high at baseline, and SBP also increased at the 2-year follow-up.

Table 5 displays the association of changes in cardiometabolic risk factors with changes in MetS status at follow-up. After adjustment for covariates, adolescents with a 1 mmHg increase in ΔSBP had a 1.07-fold risk of incident MetS at follow-up. Compared with the never group, the remission group had a greater elevation in HDL-C (ΔHDL-C, 0.61 vs. −3.67 mg/dL) and a greater decrease in TG and FPG (ΔTG, −28.12 vs. −0.35 mg/dL and ΔFPG, −8.94 vs. −2.29 mg/dL). Compared with those with persistent MetS, participants with a 1 unit increase in ΔSBP and ΔFPG had a 0.95 and 0.94-fold, respectively, lower likelihood of MetS remission after two years.

Table 6 displays the baseline prevalence, follow-up incidence density, and aHR of MetS abnormal components associated with their original status. The baseline prevalence was 24.9%, 10.6%, 21.1%, 5.5%, and 11.4% for abdominal obesity, elevated BP, low HDL-C, increased TG, and high FPG, respectively. Among the five abnormal components, low HDL-C had the highest incidence density (9.6% per year) in adolescents with an initial normal status. Abdominal obesity and low HDL-C had a greater persistent incidence density (34.3% and 36.5% per year, respectively) in participants with an original positive status. Compared with a normal status at baseline in the five MetS components, an abnormal status at baseline was associated with a higher risk of the abnormal status persisting at follow-up, with abdominal obesity and increased TG rendering a >5.0-fold risk each (aHR, 15.0 and 5.7, respectively).

## 4. Discussion

This study presents findings that demonstrate that the structure of cardiometabolic parameter clustering for adolescent MetS at baseline and follow-up were comparable. Changes in anthropometric and clinical metabolic risk factors were associated with the transformation in MetS status of adolescents after two years. Adolescents who had an abnormal MetS component at baseline were more likely to have the component be abnormal at follow-up than those who were normal for each MetS component.

MetS reflects a clustering of cardiometabolic risk parameters, which are believed to originate from the common pathophysiological mechanisms of insulin resistance [33]. In this study, a fat–BP–glucose three-factor structure for MetS was observed both in adolescents aged 12–14 years (baseline survey) and 15–17 years (follow-up survey), with analogous factor loadings and proportions of total variance explained (68.8% vs. 69.7%). In one school-based longitudinal investigation of adolescents aged 12–19 years, the overall parameter clustering structure of cardiometabolic risks did not change significantly after 3-years of follow-up [8]. These findings strengthen the argument that the mechanistic underpinning for MetS is stable during adolescence. Alternatively, the verification of a multifactor structure highlights the necessity of evaluating multisystem dysregulation in MetS using factor analysis. A risk score for MetS derived from the confirmatory factor analysis has been applied to measure the effect of the spectrum of MetS severity on T2DM and cardiometabolic disease [34,35,36].

Using four criteria to diagnose MetS in adolescents, we identified that 3.2–4.4% of adolescents in the adi-Cars cohort had incident MetS, and 52.0–61.9% of those with MetS experienced remission. Consistent with our findings, one longitudinal study that employed three MetS definitions for adolescents revealed an incident MetS rate of 3.8–5.2% and MetS remission rate of 48.6–56.1% after 2.7 years [8]. Although the four criteria for MetS abnormal components had different cutoff values, the agreement of MetS diagnosis between baseline and follow-up was similar across these criteria (*k*, 0.313–0.367), illustrating the stability of the longitudinal transformation pattern in MetS status during adolescent development. Recent prospective investigations among youth aged 9–18 years indicated that incident MetS and persistent MetS assessed by follow-up survey at two time points can substantially predict the risk of subclinical atherosclerosis (3.4-fold) and T2DM (12.2-fold) after 14–27 years [37]. The findings from a large cohort study of adults aged 40–69 years demonstrated that participants with incident MetS and persistent MetS had a 1.8- and 2.0-fold risk of developing T2DM 10 years later, respectively [38]. Thus, determining transformations in MetS status over time is a promising approach for estimating its influence on cardiometabolic disorders.

Our data revealed that intraindividual changes between baseline and follow-up in weight-related variables, HDL-C, and FPG were significant in the never MetS group, demonstrating the variability of cardiometabolic risk parameters in childhood development. After adjustment for all covariates, a high SBP increase (ΔSBP) was associated with an elevated risk of new-onset MetS (aOR, 1.07 for 1 mmHg increase; Table 5), implying that SBP elevation is critical for MetS occurrence in this population. Compared with adolescents with persistent MetS, decreases in the SBP and FPG levels were associated with an increased likelihood of MetS remission (aOR, 1.05 and 1.06, respectively, for 1 unit decrease; Table 5). Clinical studies have revealed that patients who received a short-term intensive drug treatment to lower blood glucose, BP, and cholesterol levels had a long-term reduction in the risk of T2DM and cardiovascular disorders, even after treatment cessation (known as the cardiometabolic memory phenomenon) [39]. In a community-based prospective study, adolescents in the intervention group of a triweekly exercise program and nutritional counselling were found to have a 3.0%, 18.0%, and 26.0% decrease in BMI, LDL-C, and TG levels, respectively, and a 17.0% increase in HDL-C levels, as compared with the control group [40]. Our findings underline the need for interventions among adolescents with MetS, such as dietary improvement and exercise promotion, with the aim of reducing SBP, TG, and FPG levels.

Surveillance and monitoring of the incidence and persistence of five MetS components are vital tasks in adolescent cardiometabolic health [2,15,41,42]. In our cohort, among the five MetS components, low HDL-C had the highest incidence rate (9.6% per year), whereas abdominal obesity and low LDL-C had the greatest persistence rate (34.3% and 36.5% per year, respectively). These data indicate specific risk factors that need enhanced monitoring. A combined assessment involving two longitudinal investigations demonstrated that incident MetS and persistent MetS during the transition from adolescence to young adulthood were associated with a 1.7- and 3.4-fold risk, respectively, of high carotid artery intima-media thickness, and a 4.4- and 12.2-fold of T2DM in adulthood [37]. Because the likelihood of persistence of each MetS component was higher than that for new onset (aHR, 3.4–15.0, Table 6), screening for existing abnormal MetS components can be more beneficial than preventing new-onset abnormal components.

This study had several strengths. First, a large-scale representative community-based cohort was used to prospectively assess the stability and transformation of adolescent MetS and their relation to the change of cardiometabolic risk factors. Second, our investigative framework and methodology can be adopted to other countries that wish to evaluate their own transformations in MetS status and attendant influences on cardiometabolic disorders among adolescents and adults. Third, several criteria with specific cutoff points for MetS diagnosis were simultaneously used to determine the agreement and stability for changes in MetS status over two years.

This study also had a few limitations. First, adolescents from three schools could not be followed due to the COVID-19 outbreak in Taiwan, and their data were excluded from this evaluation. However, the distributions of sex, age, urbanization level, and weight variables were comparable between the participants in the excluded and remaining schools. Second, the number of adolescents with incident, remitted, and persistent MetS was low, resulting in a wide 95% CI when polytomous logistic regression models were fitted. Larger studies are needed to confirm these results. Third, our cohort included only Taiwanese adolescents. Our findings may not be generalizable to other adolescent populations.

## 5. Conclusions

A fat−BP−glucose clustering structure underlying MetS is stable across adolescence. Increased SBP affects MetS incidence and persistence, and decreased SBP and FPG influence MetS remission after two years. Abdominal obesity and low HDL-C are two MetS components that highly persist during adolescence and are intervention targets for reducing the future risk of cardiometabolic disorders.

## Figures and Tables

**Table 1:** Demographic and cardiometabolic risk factors of the adolescent cohort measured at baseline and follow-up.

	Baseline	Follow-Up	
Factors	(*n* = 1246)	(*n* = 1155)	*p* Value ^a^
**Age, Mean ± SD**	12.6 ± 0.7	14.6 ± 0.7	<0.001
**Sex**, %			
Boy	49.0	49.1	0.979
Girl	51.0	50.9	
**Urbanization level ^b^**, %			
Level 1–2	49.9	49.3	0.946
Level 3–4	28.3	28.7	
Level 5–7	21.8	22.0	
**Cardiometabolic risk factors, Mean ± SD**			
**Anthropometric parameters**			
Waist circumference, cm	71.6 ± 11.4	74.9 ± 11.9	<0.001
Hip circumference, cm	87.6 ± 9.8	93.5 ± 9.4	<0.001
Body mass index, Kg/m^2^	20.7 ± 4.5	21.9 ± 4.8	<0.001
**Clinical parameters ^c^**			
Systolic blood pressure, mmHg	111.8 ± 13.1	114.1 ± 14.0	<0.001
Diastolic blood pressure, mmHg	64.0 ± 9.2	65.3 ± 9.0	0.001
HDL-cholesterol, mg/dL	54.1 ± 11.2	50.3 ± 10.9	<0.001
Triglyceride, mg/dL	78.1 ± 39.2	76.7 ± 36.1	0.408
Fasting plasma glucose, mg/dL	89.8 ± 11.0	88.2 ± 16.2	0.006
Glycated hemoglobin, %	5.3 ± 0.4	5.3 ± 0.6	0.650

HDL-C, high-density lipoprotein cholesterol. ^a^ *p* value for the difference in study parameters between baseline and follow-up. ^b^ The urbanization level 1 denotes the most urbanized. ^c^ Blood parameters were measured for 896 adolescents at follow-up.

**Table 2:** Exploratory factor analysis-derived factors, factor loadings, and proportions of variance explained for cardiometabolic risk factors measured at baseline and follow-up in adolescents.

Cardiometabolic Risk Factors	Baseline			Follow-Up		
	Factor Loadings (*n* = 1246)			Factor Loadings (*n* = 896)		
	Fat	BP	Glucose	Fat	BP	Glucose
[Log] Body mass index, kg/m^2^	0.901 ^a^	0.220	0.066	0.907 ^a^	0.206	0.064
[Log] Waist circumference, cm	0.899 ^a^	0.191	0.118	0.927 ^a^	0.161	0.051
Hip circumference, cm	0.875 ^a^	0.235	0.079	0.902 ^a^	0.181	0.083
[Log] Serum HDL-C level, mg/dL	−0.655 ^a^	0.175	0.058	−0.590 ^a^	0.072	−0.056
[Log] Serum triglyceride level, mg/dL	0.538 ^a^	−0.240	−0.055	0.508 ^a^	0.069	0.018
Systolic blood pressure, mmHg	0.324	0.804 ^a^	0.037	0.386	0.762 ^a^	0.091
[Log] Diastolic blood pressure, mmHg	0.142	0.835 ^a^	−0.018	0.121	0.905 ^a^	0.018
[Log] Fasting plasma glucose level, mg/dL	0.059	−0.057	0.822 ^a^	0.080	0.097	0.846 ^a^
[Log] Glycated hemoglobin, %	0.117	0.077	0.812 ^a^	0.067	−0.008	0.858 ^a^
**Eigenvalue**	3.563	1.324	1.306	3.736	1.399	1.137
**Proportion of variance explained**	36.1%	17.6%	15.2%	36.4%	16.9%	16.4%
**Cumulative proportion**	36.1%	53.6%	68.8%	36.4%	53.3%	69.7%
**Bartlett’s sphericity test, χ² (*p* value)**	5687.9	(<0.001)		4458.0	(<0.001)	
**Kaiser−Meyer−Olkin (KMO) measure ^b^**	0.782			0.794		

BP, blood pressure; HDL-C, high-density lipoprotein cholesterol; KMO, Kaiser−Meyer−Olkin. ^a^ Cardiometabolic risk factors with factor loadings >0.4 were used to interpret each factor structure. Factor loadings denote the associations between latent and observed variables. ^b^ The KMO measure >0.50 indicated that study sample was adequacy for factor analysis.

**Table 3:** Baseline and follow-up prevalences and proportions of metabolic syndrome transformation in adolescents over the 2 years of follow-up.

	Prevalence at Baseline (*n* = 896) ^a^	Incident MetS (*n* = 871)^a^	Remitted MetS (*n* = 25) ^a^	Persistent MetS (*n* = 25) ^a^	Prevalence at Follow-Up (*n* = 896) ^a^	MetS Kappa (*p* Value) ^b^
	% (95% CI)	% (95% CI)	% (95% CI)	% (95% CI)	% (95% CI)	
**IDF**	2.8	3.2	52.0	48.0	4.5	0.347
	(1.9–4.1)	(2.2–4.6)	(32.2–71.2)	(28.8–67.8)	(3.3–6.0)	(<0.001)
**TPA**	5.8	3.7	59.6	40.4	5.8	0.367
	(4.4–7.5)	(2.6–5.2)	(45.6–72.2)	(27.8–54.4)	(4.4–7.5)	(<0.001)
**JIS-Adult**	4.7	4.0	61.9	38.1	5.6	0.313
	(3.5–6.3)	(2.9–5.5)	(46.1–75.5)	(24.5–53.9)	(4.3–7.3)	(<0.001)
**IDF–TPA**	6.0	4.4	61.1	38.9	6.5	0.333
	(4.6–7.8)	(3.2–6.0)	(47.3–73.3)	(26.7–52.7)	(5.0–8.3)	(<0.001)

MetS, metabolic syndrome; IDF, International Diabetes Federation; TPA, Taiwan Pediatric Association; JIS-Adult, Joint Interim Statement of MetS for adults; IDF–TPA, the combined criteria of IDF-MetS and TPA-MetS criteria for adolescents. ^a^ At baseline, the total participants were 896; of those, 871 were MetS-negative (the candidates for incident MetS) and 25 were MetS-positive (the candidates for remitted and persistent MetS). At follow-up, the total participants were 896. ^b^ Kappa coefficient was used to examine the agreement of MetS prevalence between baseline and follow-up.

**Table 4:** Distributions and changes of cardiometabolic risk factors between baseline and follow-up for never, incident, remitted, and persistent metabolic syndrome **^a^** in adolescents.

	Never (*n* = 805)				Incident (*n* = 37)				Remitted (*n* = 33)				Persistent (*n* = 21)			
Factors	Baseline Mean	Follow- Up Mean	WP Change ^b^	*p* ^c^	Baseline Mean	Follow- Up Mean	WP Change ^b^	*p* ^c^	Baseline Mean	Follow- Up Mean	WP Change ^b^	*p* ^c^	Baseline Mean	Follow- Up Mean	WP Change ^b^	*p* ^c^
**BMI**	19.93	21.09	1.16 *	<0.001	26.76	28.59	1.83 *	<0.001	27.21	28.12	0.91	0.187	30.80	31.91	1.12	0.151
**WC**	69.90	72.96	3.06 *	<0.001	85.51	91.06	5.55 *	<0.001	87.43	90.06	2.63	0.187	95.30	98.46	3.16	0.151
**SBP**	111.04	111.97	0.94	0.065	119.38	132.57	13.19 *	<0.001	121.85	122.06	0.21	0.932	126.90	137.76	10.86 *	0.039
**DBP**	63.68	64.43	0.75	0.065	67.24	72.62	5.38 *	<0.001	70.97	69.06	−1.91	0.532	71.47	76.19	4.72	0.159
**HDL-C**	55.00	51.33	−3.67 *	<0.001	47.03	41.08	−5.95 *	<0.001	42.43	43.04	0.61	0.774	39.88	37.71	−2.16	0.151
**TG**	73.38	73.03	−0.35	0.763	99.78	109.22	9.43	0.315	123.39	95.27	−28.12 *	<0.001	128.71	131.43	2.71	0.807
**FPG**	89.48	87.20	−2.29 *	<0.001	89.68	89.43	−0.24	0.872	95.97	87.03	−8.94 *	<0.001	104.48	126.52	22.05	0.151
**HbA1c**	5.29	5.28	−0.004	0.763	5.31	5.28	−0.03	0.689	5.30	5.31	0.02	0.887	5.75	6.42	0.67	0.151

WP, within-person; BMI, body mass index; WC, waist circumference; HDL-C, high-density lipoprotein cholesterol; TG, triglyceride; SBP, systolic blood pressure; DBP, diastolic blood pressure; FPG, fasting plasma glucose; HbA1c, glycated hemoglobin. ^a^ IDF–TPA criteria were used to determine adolescent metabolic syndrome. ^b^ The mean level for within-person change in the cardiometabolic risk factors between baseline and follow-up. Here, * denoting *p* < 0.05 for the pair difference between baseline and follow-up. ^c^ *p* value for WP change was obtained from the linear mixed model adjusted for sex, age, and urbanization level and was adjusted for false discovery rate.

**Table 5:** Adjusted associations of the changes in cardiometabolic risk factors over 2 years of follow-up with incident, remitted, and persistent metabolic syndrome **^a^** in adolescents.

Within Person Change ^b^	Never (*n* = 805)		Incident (*n* = 37)		Remitted (*n* = 33)		Persistent (*n* = 21)		Remitted vs. Persistent
	Mean (SD)	aOR (Ref.)	Mean (SD)	aOR ^c^ (*p* Value ^d^)	Mean (SD)	OR ^c^ (*p* Value ^d^)	Mean (SD)	OR ^c^ (*p* Value ^d^)	aOR ratio ^c^ (*p* Value ^d^)
**ΔBMI, Kg/m^2^**	1.16 (1.71)	1.0	1.83 (1.90)	0.98 (0.949)	0.91 (2.98)	1.08 (0.949)	1.12 (3.31)	1.09 (0.949)	0.99 (0.949)
**ΔWC, cm**	3.06 (6.11)	1.0	5.55 (6.67)	1.05 (0.784)	2.63 (9.15)	0.99 (0.784)	3.16 (9.03)	1.02 (0.784)	0.97 (0.784)
**ΔSBP, mmHg**	0.94 (13.47)	1.0	13.19 (13.65)	1.07 * (<0.001)	0.21 (14.54)	1.01 (0.680)	10.86 (18.09)	1.07 * (0.004)	0.95 * (0.039)
**ΔDBP, mmHg**	0.75 (10.80)	1.0	5.38 (9.16)	0.99 (0.751)	−1.91 (11.50)	0.97 (0.751)	4.72 (14.98)	0.99 (0.751)	0.98 (0.751)
**ΔHDL-C, mg/dL**	−3.67 (7.86)	1.0	−5.95 (6.45)	0.97 (0.310)	0.61 (6.43)	1.08 * (0.013)	−2.16 (6.41)	1.03 (0.365)	1.05 (0.310)
**ΔTG, mg/dL**	−0.35 (33.12)	1.0	9.43 (49.13)	1.01 (0.253)	−28.12 (41.89)	0.98 * (<0.001)	2.71 (52.18)	0.99 (0.427)	0.98 (0.095)
**ΔFPG, mg/dL**	−2.29 (10.39)	1.0	−0.24 (9.33)	1.02 (0.209)	−8.94 (10.80)	0.96 * (0.014)	22.05 (65.38)	1.02 (0.173)	0.94 * (0.011)
**ΔHbA1c, %**	−0.004 (0.29)	1.0	−0.03 (0.32)	0.98 (0.968)	0.02 (0.31)	0.77 (0.935)	0.67 (1.97)	2.21 (0.474)	0.35 (0.474)

SD, standard deviation; Ref., reference group; aOR, adjusted odds ratio; BMI, body mass index; WC, waist circumference; HDL-C, high-density lipoprotein cholesterol; TG, triglyceride; SBP, systolic blood pressure; DBP, diastolic blood pressure; FPG, fasting plasma glucose; HbA1c, glycated hemoglobin; *, *p* < 0.05. ^a^ IDF–TPA criteria were used to determine adolescent metabolic syndrome. ^b^ The within-person differences in the cardiometabolic risk factors (follow-up values minus baseline values, denoted as Δ). ^c^ aORs were obtained from polytomous logistic regression models adjusted for sex, age, urbanization level, and covariates in the Table. ^d^ *p* values were adjusted for false discovery rate.

**Table 6:** Baseline prevalences, follow-up incidence densities, and adjusted hazard ratios of abnormal components of metabolic syndrome associated with initial status over 2 years of follow-up in adolescents.

	Abnormal Components of MetS ^a^									
Factors	Abdominal Obesity		Elevated BP		Low HDL-C		Increased TG		High FPG	
**Baseline**										
No. of positive	310		132		263		69		142	
Prevalence	24.9%		10.6%		21.1%		5.5%		11.4%	
**Follow-up**	No	Yes	No	Yes	No	Yes	No	Yes	No	Yes
Participants at follow-up, no. **^b^**	862	293	1026	129	696	200	851	45	777	119
Person-year, year	1886.7	634.8	2242.6	279.0	1484.8	430.2	1814.9	100.0	1657.3	257.7
** Abnormal component**										
No	808	75	918	68	553	43	823	35	736	97
Yes	54	218	108	61	143	157	28	10	41	22
** Incident density, per year ^c^**	2.9%	34.3%	4.8%	21.9%	9.6%	36.5%	1.5%	10.0%	2.5%	8.5%
** aHR (95% CI) ^d^**	1.0	15.0	1.0	4.0	1.0	3.4	1.0	5.7	1.0	3.8
		(11.0–20.3)		(2.9–5.5)		(2.7–4.3)		(2.6–12.2)		(2.1–6.7)

BP, blood pressure; HDL-C, high-density lipoprotein cholesterol; TG, triglyceride; FPG, fasting plasma glucose; MetS, metabolic syndrome; aHR, adjusted hazard ratio. ^a^ IDF–TPA criteria were used to determine the abnormal components for MetS. ^b^ Participants who were evaluated for obesity and BP were 1155 adolescents and for HDL-C, TG, and FPG were 896 adolescents at follow-up. ^c^ Incidence density was used to assess the status of new-onset MetS components in the follow-up survey. ^d^ aHRs were adjusted for sex, age, and urbanization level.

## Data Availability

The data are not publicly available due to privacy restrictions.

## Supplementary Materials

The following are available online at https://www.mdpi.com/article/10.3390/nu14040744/s1. Table S1: Diagnostic criteria for the determination of metabolic syndrome defined by IDF, TPA, JIS-Adult, and IDF–TPA. Figure S1: Scree plots for exploratory factor analysis of 9 metabolic risk variables measured at (A) baseline and (B) follow-up.

## Abbreviations

**aHR**, adjusted hazard ratios; **adi-Cars**, adiposity–cardiovascular–disease axis; **BP**, blood pressure; **BMI**, body mass index; **DBP**, diastolic blood pressure; **EFA**, exploratory factor analysis; **HbA1c**, glycated hemoglobin; **HDL-C**, high-density lipoprotein cholesterol; **HC**, hip circumference; **IDF-TPA**, International Diabetes Federation and Taiwan Pediatric Association; **JIS-Ad**, Joint Interim Statement for adult MetS; **KMO**, Kaiser-Meyer-Olkin; **MetS**, metabolic syndrome; **FPG**, fasting plasma glucose; **SBP**, systolic BP; **T2DM**, type 2 diabetes; **TG**, triglyceride; **WC**, waist circumference.
