# Supplementary material for: Stability and Transformation of Metabolic Syndrome in Adolescents: A Prospective Assessment in Relation to the Change of Cardiometabolic Risk Factors
Source: Nutrients. 2022 Feb 10;14(4):744. doi: 10.3390/nu14040744 (PMC8875515; doi:10.3390/nu14040744)
Supplement: Supplementary file 1 [file nutrients-14-00744-s001.zip › nutrients-1546131-supplementary.pdf]

Supplementary Table S1. Diagnostic criteria for the determination of metabolic syndrome defined by IDF, TPA, JIS-Adult, and IDF–TPA

MetS criteria	MetS abnormal components					Diagnostic criteria for MetS
	Abdominal obesity	Low HDL-C	Increased TG	High FPG	Elevated blood pressure	
**IDF (for 10-18 yrs) [23]**	WC ≥90 percentile (or adult cutoff if lower)[1]	<40 mg/dL (10-15 years); <40 mg/dL in males, <50 mg/dL in females (16-18 years)	≥150 mg/dL	≥100 mg/dL (or diagnosed T2DM before)	SBP ≥130 / DBP ≥85 mmHg (or antihypertensive drug treatment)	**Central obesity + 2 other RCs**
**TPA (for 8-18 yrs) [24]**	BMI >95 percentile for age-sex-specific groups[2]	<40 mg/dL in males, <50 mg/dL in females	≥150 mg/dL	≥100 mg/dL (or diagnosed T2DM before)	SBP ≥130 / DBP ≥85 mmHg (or SBP/DBP > 95 percentile for age-sex-specific groups)	**Central obesity + 2 other RCs**
**JIS-Ad (for Asians ≥18 years) [6]**	WC ≥90 cm in males, WC ≥80 cm in females[3]	<40 mg/dL in males, <50 mg/dL in females	≥150 mg/dL	≥100 mg/dL (or diagnosed T2DM before)	SBP ≥130 / DBP ≥85 mmHg (or antihypertensive drug treatment)	**Any 3 or more RCs**
**IDF–TPA (for 12-18 yrs) [15,26]**	WC ≥90 percentile (or adult cutoff if lower)[1], or BMI >95 percentile for age-sex-specific groups[2]	<40 mg/dL in males, <50 mg/dL in females	≥150 mg/dL	≥100 mg/dL (or diagnosed T2DM before)	SBP ≥130 / DBP ≥85 mmHg (or SBP/DBP > 95 percentile for age-sex-specific groups, use antihypertensive drug)	**Central obesity + 2 other RCs**

Abbreviation: MetS, metabolic syndrome; HDL-C, high-density lipoprotein cholesterol; TG, triglyceride; FPG, fasting plasma glucose; IDF, International Diabetes Federation; TPA, Taiwan Pediatric Association; JIS-Ad, Joint Interim Statement of MetS for adults; WC, waist circumference; BMI, body mass index; SBP, systolic blood pressure; DBP, diastolic blood pressure; T2DM, type 2 diabetes; RC, risk component.

[1] If BMI is > 30kg/m², abdominal obesity can be assumed, waist circumference does not need to be considered.

[2] The cut-off points of abdominal obesity for age-sex-specific adolescents are defined by the TPA.

[3] The WC thresholds (i.e., WC ≥90 cm in males and ≥80 cm in females) suggested for Asian adult abdominal obesity were applied.

[4] The combined MetS criteria were used to include adolescents who meet the IDF or TPA criteria.

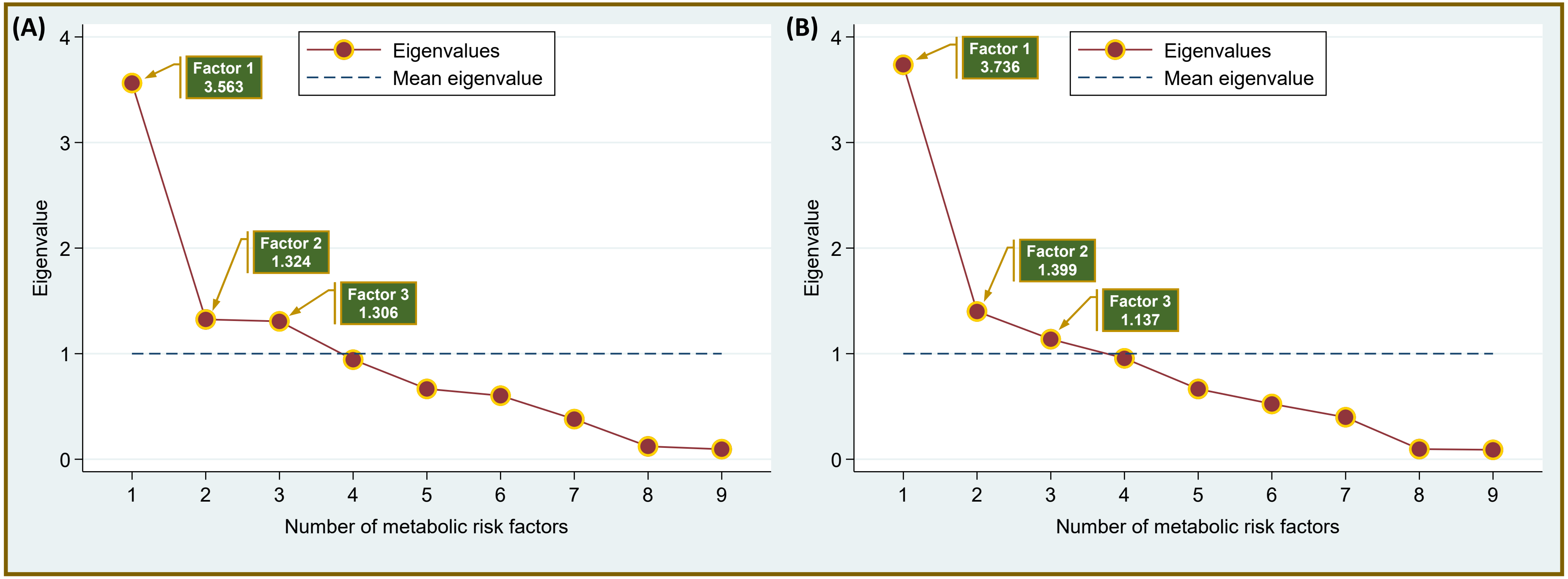

Supplementary Figure S1. Scree plots for exploratory factor analysis of 9 metabolic risk variables measured at (**A**) baseline and (**B**) follow-up, respectively.

Note: There were three factors in that their eigenvalues were >1 and surpassed the break in the scree plots at baseline and follow-up, respectively.
